# Making and Unmaking a “Bactericidal” Organism: Sterile Surgical Maggots and Organic Antiseptics in Inter-War America

**DOI:** 10.1093/jhmas/jrac025

**Published:** 2022-06-28

**Authors:** Tom Quick

**Affiliations:** Maastricht University, The Netherlands

**Keywords:** animal, interwar, surgery, infection, hygiene

## Abstract

This article charts the popularization of and eventual disengagement with an approach to wound infection control centered on the medical efficacy of living beings (maggots) in 1920s and 1930s America. Baltimore surgeon William Stevenson Baer successfully drew on his wartime experience to promote the use of sterile or “surgical” maggots in the treatment for deep-seated bone infection at this time. This article situates the practices he promoted in the context of President Herbert Hoover's contemporary establishment of the “associative state,” thereby drawing out the relevance of the latter to medical governance. In so doing, it conveys an image of the development of early twentieth-century infection control that contrasts with narratives centered on medically productive aspects of biomedical industrialization and specialization. The incorporation of surgical maggot research and evaluation within the United States Department of Agriculture played a critical role in the subsequent abandonment of the use of maggots as medical aides. Hoover's orientation of this institution towards the support of large-scale private enterprise helped motivate efforts to synthetically replicate (and thereby displace) maggots' medical efficacy. Ultimately, maggot-based therapeutics proved incompatible with conditions created by the orientation of American governmental institutions towards corporate subsidy rather than the support of individuals.

On 17 April 1930, ex-military surgeon and head of orthopedic surgery at Johns Hopkins University William Stevenson Baer stood in front of the United States House of Representatives Committee on World War Veterans' Legislation and explained his plan for the rehabilitation of many of the long-term convalescent patients in America's hospital wards.^1^ His vision was a counter-intuitive one. The problem, as outlined by Republican Senator for South Dakota Royal C. Johnson, was that thousands of soldiers who had served in the trenches during the European War continued to be unable to join the workforce. In taking up expensive hospital beds and their need for resource-intensive, ongoing treatment, these ex-soldiers were both draining charitable and governmental resources and failing in their duties as breadwinners.^2^ In this instance, the causes of such personal inadequacy were tragically obvious. The patients were suffering from deep-seated bone infections, or osteomyelitis. Wounds originally picked up on the battlefields of Europe continued to ooze with pus and blood, preventing both pain-free activity and general constitutional improvement. Yet, Baer expressed new hope for military men crippled by these long-term injuries. Their wounds, he announced, no longer needed to be subject to constant sluicing with antiseptics, routine surgical removal of necrotic material, and endlessly piped irrigation: they could instead be filled with maggots. Blowfly larvae, he contended, had been shown to eat away at such wounds, ingesting only infected parts and leaving space for the growth of healthy reparative tissue.

That a senior American surgeon began extolling the wound-healing properties of fly larvae at this time will perhaps come as a surprise to historians of inter-war American medicine. After all, flies in general had come to be identified as a particularly pernicious public health threat during the first decades of the twentieth century. They were by 1930 well-recognized as carriers of the all-pervasive germs responsible for tuberculosis, cholera, and typhoid (amongst many other diseases). Their apparent reliance on shit both for food and as a site of reproduction cast these animals even more firmly as enemies of healthy existence at a time when American “antisepticonsciousness” was at its height.^3^ Amongst surgeons, intensive disinfectant regimen and absolute asepsis in the operating theater had become established, broad-based ideals to which it was assumed all should aspire.^4^ Yet, the prospect of “again mak[ing] useful citizens of otherwise permanent invalids” outweighed any hygiene qualms Baer had.^5^ Indeed, as he reassured his audience, the maggots that he had been using were not the dirty creatures of public health nightmare. They had rather been uniquely purified, cleansed, and generally made suitable for placing on a person's body. They were in fact what he called a “violable antiseptic,” comparable with such well-known surgical disinfectants as carbolic acid, sodium hydroxide, and the then-famous Carrel-Dakin (sodium hypochlorite) solution.^6^

The Committee's enthusiastic response to Baer's proposal set in motion a series of events that have by-and-large disappeared from the annals of American medicine. Baer, in a story that would be repeated ad nauseam in articles and speeches on maggot therapy, recalled that his initial interest had been piqued by his wartime experience witnessing the return to camp of two badly injured soldiers with maggot-infested wounds. On being cleaned out, the wounds themselves had been in surprisingly good condition, despite the fact that the soldiers had lain on the battlefield for several days.^7^ Baer's unconventional approach grew in popularity during the 1930s, accompanied by numerous popular and academic publications, increasing professional recognition amongst surgeons, and a government-led research program. A bibliography of 1936 cites 148 academic articles published on the topic in the previous fifteen years. The majority of these were contributions to reputable American medical journals, though also included a small number of studies in French, German, and Spanish.^8^ Yet the practice had been almost entirely forgotten three decades later, only gaining renewed remembrance in medical circles from the mid-1980s, as fears around antibiotic resistance began to attract serious academic attention.^9^ In elucidating how and why it was that such a unique and apparently effective approach to wound care could have been so rapidly abandoned, this article concentrates exclusively on the former period. In so doing, it highlights an historical episode in which, despite the contemporary tendency towards the exclusion of macroscopic organic therapeutics, American medical scientists and practitioners came to accord healing capacities to living, non-human beings. The article further considers the extent to which such accordance was sustainable within the particular political and economic conditions prevalent in the US during the 1930s.

There is of course one obvious reason why maggot therapy has so rarely been mentioned in narrative accounts of twentieth-century infection control, and that is that the practice sits decidedly awkwardly with the almost exclusive identification of post-1935 antiseptic medicine with the development and application of chemotherapeutic and microbiologic technologies. The undoubted triumphs of antibiotics during the middle decades of the twentieth century gave an impression that pharmaceutical big science had alone been responsible for the emergence of effective means of controlling microbes — a theme also evident in the literature on “biologics” and contemporary biochemical interest in abiosis, to which antibiotics can trace a direct lineage.^10^ This literature identifies a relatively uncomplicated transition from the then-emerging biochemical industrial discourse around the extraction and synthesis of corporeally active substances (typified by the German concept of *Wirkstoffe*) and the turn to microbially-manufactured antiseptics (the term antibiotic only found purchase after 1940) from the late 1930s.^11^ It is not the purpose of this article to deny the broad validity of this narrative. I do however aim to demonstrate that, at least at the level of surgical practice, the historiographical picture relating to the use of organism-based approaches to infection control is more complex than studies centered exclusively on the increasing contemporary association of medical scientific developments with large-scale industry allow.

The (albeit brief) American fascination with maggot therapy highlights that in their efforts to repair and enhance the human body, practitioners turned not just to highly technical laboratory-centered processes, but also to more mundane, macroscopic forms of life. Maggot therapy as articulated by Baer had more in common, both in practice and in institutional location, with efforts directed at so-called “biological control” of crop pests such as were then being promoted within the US Department of Agriculture (USDA) than it did with the creation and application of mysteriously efficacious micro-biochemical matter.^12^ Maggot therapy in this respect helped constitute a therapeutic culture that centered on the action and interaction of whole living organisms, holistic visions of social exchange, and even, in some articulations, on relations between only distantly related living species.^13^ Proponents of maggot therapeutics participated in a culture in which not only the products of organic beings, but also their by-and-large unadulterated bodies, were seen as a scientifically legitimate means by which pathogens might be brought under control. This article thereby indicates the presence during the 1930s of a broader range of life-based therapeutics than have been acknowledged in studies focused solely on biologics and their antibiotic successors.

More generally however, this article shows that at least some attempts to establish whole organism-centered approaches to the control of disease were undermined by the broader political and economic climate of the period. The destiny of maggot-based therapeutics came to be determined by a US government research program that was firmly oriented towards the promotion of large-scale industrialization. Recent work in the history of science has called for renewed engagement with historical intersections between science and capitalism, and the structures and processes through which they are mutually “entangled.”^14^ The inter-war period in the US offers particularly fertile territory for such investigation, constituting as it does a moment in which relations among the federal government, private corporations, and scientific (in this case biomedical) practice came to be hotly contested. The emergence during the 1920s of sustained efforts to harness federal administrative capacities to direct and facilitate corporate ends is particularly notable in this regard. Under the administration of President Herbert Hoover, American businesses came for the first time to be seen as legitimate objects of direct federal support.^15^ Yet, whilst the critical role of wealthy American charitable foundations in encouraging the emergence of self-consciously scientific medical research practice is now well documented, less is known of the degree to which Hoover's consciously corporation-supporting “associative state” influenced 1930s medical culture.^16^ Except in their regulatory capacity, institutions of state are rarely portrayed as having molded relations amongst scientists and medical practitioners at this time.^17^ As a case study in which a federal research program directly supported both the activities of individual medical practitioners and medicine-focused corporations, this article thereby fosters a more complete understanding of the influence of Hoover's voluntaristic vision over medical practice during the 1930s. Most notably, it shows how the then-emergent science of whole-organism therapeutics, in which insects and other animals appeared as potential aids in the promotion of human well-being, waned in the face of sustained contact with governmental and corporate organizations oriented towards the development of technically complex, patentable products that could be mass produced in laboratories and factories. Ultimately, the maggots employed for therapeutic purposes by Baer would be reduced to yet another source from which ever more commercially exciting biologics might be identified, manufactured, and applied.

Whilst the article begins by focusing on Baer's initial efforts to establish maggot therapy as a practical and above all scientifically plausible technique, its central focus is on the way Baer's original holistic vision came to be eroded by both the governmental impulse towards the support of large-scale commerce and pharmaceutical companies themselves. As sterile or surgical maggots came to be seen as significant objects of interest within the US government — specifically at the same USDA Bureau tasked with administering biological control experiments — they also came paradoxically to be ever more excluded from the surgical ward.^18^ Similarly, an initial institutional focus on governmental collaboration with surgeons rapidly gave way to a more general concern with serving the needs of manufacturers of pharmaceuticals. Although maggot therapy can be said to have participated, alongside many microbiologic products, in the turn towards the utilization of life for therapeutic ends out of which antibiotics emerged during the 1940s, the commercial culture of the time ultimately undermined its viability as a medical practice. It was the unsuitability of maggots as medical commodities, rather than any considered judgment as to their efficacy in infection control, which led to their therapeutic abandonment during the middle decades of the twentieth century.

## Holism, Anti-Listerism, and the Medical Maggot

Hoover's presidency came to be defined by a single outstanding event and its consequence: the Wall Street crash and subsequent depression. His intransigence regarding what he considered unacceptably communistic forms of state support in the face of a rapidly spiraling economic collapse contrasted with his more general optimism that a combination of a minimalist state, individual self-reliance, and a market left to its own devices would restore American economic pride. For Hoover, the federal government's role was not to stand in for moribund American businesses, but rather to facilitate the emergence of economic modernization, in much the same manner that the Commerce Department had done under his leadership during the 1920s. There, Hoover presided over the emergence of a new model of state function in which, rather than providing services to the populace directly, government departments acted as clearing-houses and catalysts for commercial operations. They achieved this primarily through the sharing of statistical information between firms, organizing meetings to coordinate production, guiding businesses through the development of novel manufacturing processes, and providing access to state-developed patents. Hoover and his aides notably envisioned the federal government withering away once the constitution of a self-regulating “cooperative system” of voluntaristic and commercial “organisms” had been achieved.^19^ An engineered system of balanced economic interests could he believed foster cooperation among all kinds of private entities great (such as corporations and charities) and small (such as family businesses and individual breadwinners).

This broad-based vision of a state-facilitated, emergent balance between independent and mutually reliant bodies resonated with similar understandings of both agricultural and medical practice. Historians have shown how metaphors of economic balance were inextricably linked with corporeal conceptions at this time.^20^ Whilst they did not always subscribe to Hoover's vision, many holistic thinkers in America were committed to the integration of laboratory investigation with a clinical practice properly rounded with humanistic knowledge and social scientific awareness. The body was for them a conglomerate of independently acting, mutually dependent organs connected via fluidic flows and modulated by chemical regulators. For the most broadly influential American holist Lawrence J. Henderson, medical practitioners and patients constituted a system of interacting parts operating within a wider institutional context. Successful treatment and institutional efficiency alike required facilitation via the flow of bodies and promotion of intellectual exchange.^21^ Similarly, Johns Hopkins University physician George Canby Robinson oversaw the “organic association” of the New York Hospital and the Cornell Medical College during the late 1920s.^22^ The Hoover administration thus emerged in conjunction with an intellectual connection of body and economy that not only drew on holistic physiological metaphor, but also encouraged the organization of medical practice around the integration of mutually concerned institutions. By considering and where necessary intervening in body and mind simultaneously, many American physicians thus hoped to foster the emergence of a self-reliant state in which individuals, like the country of which they were a part, would both maximize their natural productive capacities and gain sufficient strength to ward off future threats.

Surgery was the paradigmatically reductionist discipline in opposition to which inter-war medical holists set their stalls. Ideals of specialized, organ-specific knowledge, division of medical labor, and standardized technique over individual skill had found particular surgical favor.^23^ Nevertheless, for the undoubtedly many surgeons who remained skeptical of unbounded scientific rationalization, the European War had been a reminder that practices perfected in the calm of civilian hospital wards and cloistered laboratories were not necessarily appropriate for all eventualities. For example, implementation of the highly technical procedures of asepsis had seemed virtually impossible in the trenches. Despite attempts to introduce cleaner conditions near the front, army hospitals saw already-serious injuries compounded by notoriously deadly infections such as tetanus and gas gangrene. It seemed counterproductive to follow guidelines developed in gentler times and places in these circumstances. As surgeons despaired of established approaches to microbial management, a multitude of novel disinfectants, including mercury salts, bleaching powder mixed with boric acid, and an antiseptic paste known as BIPP (bismuth, iodoform and liquid paraffin) had been trialed on wounded soldiers. Many surgeons even began to doubt basic Listerian principles, with figures such as British surgeon Almoth Wright and his assistant Alexander Fleming arguing against any use of strong chemical antiseptics.^24^ Whilst a need for complete debridement of dirt-infused injuries was widely agreed upon by 1918, little concord had been reached as to the most practical means of dealing with wound infection in general.

The apparent triumph of the highly technical and specialized Carrel-Dakin method of radical aseptic debridement and careful irrigation of wounds in the years following disarmament was then tempered by a sense that specialized scientific surgery had not been able to solve every problem of wound care. For already infected wounds, it was often difficult to identify every last site of infection. Many people languished for years fighting recurrently suppurating wounds and undergoing yet further experimental operations. So despite a general improvement in recovery rates since the late nineteenth century, the persistence of long-term cases invited surgical dissent. By the end of the 1920s, two new techniques — the so-called Orr method and Baer's maggot therapy — had emerged that became emblematic of a new space that had opened up for surgical holists.^25^ Whilst aspects of the Orr method (which involved the rapid encasement of bone injuries in plaster cast, accompanied by a minimalist approach to microbial management) remain iconic within orthopedic surgery today, it is the less well-known maggot therapy that had the greatest influence on subsequent approaches to healing already infected wounds.

Baer's understanding of the human body as an integrated whole thereby illustrates the more general intersection among holism, military surgery, and American political concern with returning incapacitated citizens to economically functional life during the 1920s.^26^ His holistic approach to bodily ailment was not confined to wound care. At the 1929 meeting of the Inter-State Medical Association in Detroit, during the talk in which he first described the maggot technique to a professional audience, Baer professed equal if not greater concern with a similarly debilitating condition, that of arthritis deformans (chronic arthritis). This he split into “infectious,” “atropic” and “hypertrophic” types. Infectious arthritis especially was “not only a local [disease], but… a constitutional one” in which infection had “gone through the body.”^27^ He went on to similarly characterize exclusive appeal to antisepsis and asepsis in wound care as problematic. Over-use of the stronger chemical antiseptics, he claimed, “change[s] certain [bodily] principles which are trying to make that wound heal,” resulting in a situation in which “wound[s] get well in spite of us rather than with our present treatment.”^28^ Thus he admonished surgeons to take cognizance of the whole bodies of patients, attending to the ways in which different regions interacted and their capacities for self-regeneration.

The notion that bodies contain self-healing principles had become a significant object of holistic medical research during the first decades of the twentieth century. Much of the impetus for this had come came from Europe.^29^ In England for example, Wright and Fleming utilized “vaccine therapy,” wherein bacterial cultures were injected into patients’ bodies with the aim of facilitating their presumedly inherent natural defenses.^30^ Though he did not reference such therapies directly, Baer interpreted the action of maggots in terms of a kind of bodily stimulation. Though he was loath to make a definite statement in this regard, it seemed that they did at least have “subtle biochemical effects…and perhaps cause also a constitutional reaction inimical to bacterial growth.”^31^ The health-inducing partnership between human and maggot bodies also resonated in some ways with the political ideal of America as a country of autonomous private entities working in harmonious unison towards a greater good. Properly managed, maggots were efficient consumers of necrotic wound tissue that “like bone as dogs do,” hunting fragments and bringing them up to the wound's surface.^32^ Human bodies, in response, seemed to Baer to emit some kind of substance that aided their own healing: there was “something formed between a maggot and a human body” that could be “an enzyme.”^33^ The suggestion was immediately latched onto by science journalists and newspaper reporters alike.^34^ Indeed, prospects for the development of a disinfectant “enzyme therapy” would gain popular plausibility in the light of Charles Avery and Rene Dubos's contemporary announcement that they had discovered a bacterially-produced substance that could “decapsulate” the type III pneumococcus specifically responsible for lobar pneumonia.^35^

Baer related that his awareness of the necessity of using only sterilized maggots had arisen from practical problems he had encountered during his early trials at the hospital in Baltimore from which many of his initial patients were drawn. He had, he suggested, begun naively, attracting flies from the surrounding environment using putrid meat and collecting the eggs that they laid on it. These he had transferred once hatched directly into patient's wounds.^36^ Despite seemingly universally positive responses, two dramatic incidents had turned his attention to maggot bodies as potential sources of bacterial pollution. Firstly, one patient's wound was found to be infected with the highly dangerous gas gangrene. Fortunately, the notoriously fast-acting bacterial infection had been caught early and further maggot treatment had it seemed helped avoid its usual deadly course. Believing that a maggot must have carried the gas bacillus into the wound, Baer had consequently adopted a policy of immersing his environmentally-sourced larvae in a disinfectant solution of bichloride of mercury. Despite this precautionary measure, however, a second infection was soon detected in another patient, this time of tetanus (lockjaw), from which the sufferer only recovered after what Baer described as a “hard fight.”^37^ Again identifying maggots as the source of the infection, Baer decided it was necessary to guarantee that all further implantations were free of microbial invaders. He would find a means of raising completely disinfected larvae in captivity, and so remove any chance that they might come into contact with pathogenic agents.^38^

Baer's concern with maggots rather than surgical disinfection thereby displaced the conventional focus of aseptic practice from the surgical theater and wound, onto an external, reputationally unhygienic living being that could in principal be utilized by anyone. In purifying his maggots, he aimed to reconcile their status as dirty animals with surgeons' overarching suspicion of all material considered foreign to hospital wards and operating theaters. The fully-developed technique nevertheless focused on the disinfection of blowfly eggs rather than the maggots themselves. Baer and his co-workers assumed that by ensuring that eggs were entirely free of pathogens, and providing an as-far-as-possible microbe-free environment for their cultivation, it would be possible to guarantee a surgically-appropriate level of cleanliness in the animals. Thus eggs were immersed in the aforementioned bichloride of mercury and hydrochloric acid, and raised on “sterile synthetic food composed of [autoclaved] liver, agar, yeast, and honey”: “the sterile maggot” was “laid down on a sterile medium.”^39^ Hungry young maggots would subsequently be introduced into infected regions of patients’ bodies in batches. Each implantation was kept in place by a wire mesh cage, eating away at necrotic material until they either matured enough to pupate or ran out of food. A particularly beneficial feature of the blowfly maggots he used, Baer emphasized, was that they did not eat healthy tissue, a circumstance that removed any requirement for onerous monitoring regimes.^40^ Patients’ bodies, in combination with maggots, would thereby be allowed to recover their self-regulating capacities.

Given the widespread assumption that maggots were irretrievably infested with pathogens that should as far as possible be excluded from surgical practice, it is unsurprising that the treatment immediately evinced hostile reactions within the profession. A colleague of Baer's recalled that it was initially seen as a “colossal medical joke…too ridiculous even to be taken seriously.”^41^ Moreover, regardless of any hygiene anxieties they might prompt, maggot application seemed to some yet another addition to an already long list of unreliable or over-complicated procedures.^42^ Most objections, however, remained focused on the status of maggots as disease vectors. New York surgeon Walton Martin dismissed Baer's claims out of hand, emphasizing that flies in all their forms had “justly been considered the most dangerous of all the disseminators of pathogenic microorganisms.”^43^ Echoing professional skepticism, one early newspaper report acerbically suggested that “probably if we had but yellow butterflies or green pickles in the same wound, they would have healed anyhow.”^44^

Yet for a small but growing band of supporters, the results of maggot therapy spoke for themselves. Baer's assistant Stanton Knowlton Livingston claimed that, “Dr. Dean Lewis, professor of surgery in the Johns Hopkins Hospital” had deemed the discovery “comparable with that of Lord Lister.”^45^ Another physician called it “probably the most outstanding development in medical science since the discovery of Salvarsan.”^46^ In many cases superlatives were accompanied by detailed claims to efficacy. In addition to single-case reports, larger cohort studies began to emerge which, while they did not engage with contemporary moves towards statistically sophisticated control trials, lent an air of mathematical rigor to Baer's initial results. Maggots began to be applied to non-osteomyelitic wounds, too.^47^ To give just one example, Walter A. Coakley and Samuel Kline reported on a severe case of an “acute suppurative and gangrenous appendix” in which almost every possible approach had been tried, including “electrocautery”-induced excision, “treatment with Dakin solution, staphylococcus jellies and silver nitrate solution” and further “extensive debridement,” as well as application of “cod-liver oil pastes, chlorinated lime, zinc peroxide, [and] high vitamin diets.” At a loss after eight months' experimentation, “1000 maggots” had been “poured into the wound,” following which rapid healing commenced.^48^ As surgeons became more confident, they began to compare maggot therapy to other disinfectant techniques. Joseph Buchman of Brooklyn, New York, found “results…superior to those of the Carrel-Dakin method” and noted that although the Orr treatment also appeared effective in similar kinds of case, the latter was nevertheless “objectionable because of its offensive odor.”^49^ This gradual accumulation of positive reports, combined with an associated literature addressing the technical problem of maggot purification, reflected a growing willingness to experiment. A 1935 survey reported that 585 surgeons from every state had among them treated a total of 5684 cases.^50^

Some even considered maggot therapy to have implications for surgical practice more generally. In line with his holistic views, Baer declared that “Lord Lister never intended” asepsis to be pursued to the exclusion of other cases, noting that “we do a great deal of harm in the treatment of infected wounds by means of chemicals.”^51^ Such denouncements were frequently accompanied by the characterization of maggots themselves as antiseptic agents. Thus Livingston stated that “maggots are living antiseptics…especially useful in…molecular necrosis.”^52^ Portrayals of sterile maggots as comparable to synthetic antiseptic technologies fostered commercial interest, and with it increasingly breathless descriptions of the newly reputationally and physiologically laundered beings. A representative of Lederle Laboratories of Pearl River, New York, the first commercial outfit to attempt large-scale sterile maggot production, declared the therapy to be “fascinating to the medical profession because it invokes an entirely new principle…a biological in contrast to a chemical antiseptic.”^53^ Alongside enzyme therapy, maggot healing of wounds seemed in Lederle's literature to be the only proven, accessible, organism- (rather than synthetic chemical-) centered approach to combatting infection.

Yet the specific mechanism by which maggots assisted in wound healing remained unclear. Apart from Baer's suggestion that they brought forth the body's natural capacity for healing, emphasis was placed on the mechanical and chemical action of eating and digestion. At least initially, it seemed to many that it was through simple consumption of necrotic material that wounds were cleansed.^54^ Even a short excursion into entomological science revealed that the actual processes of maggot ingestion were by no means fully understood. During the late nineteenth century, pioneering French entomologist Jean-Henri Fabre had claimed that blowfly maggots did not (as had previously been presumed) consume solid pieces of flesh, but rather like flies themselves secreted a digestive fluid from their mouths and sucked up the resulting liquefied matter. This led some to suggest that surgical maggots were removing necrotic tissue “by means of…predigestion, or liquefaction.”^55^ Others believed that maggots somehow contributed to a transformation of wounds from acid to alkali, creating a chemically inhospitable environment for further bacterial growth.^56^ Some even entertained the possibility that it was not the digestion of infective material that had the greatest impact on the wound, but rather the nature of larval excrement.^57^ Despite their differences, all of these proposals shifted attention away from Baer's initial interest in interactions between human bodies and maggots and towards the idea that healing was caused by some hidden substance within maggots alone. This tendency had the effect of encouraging medical analysts to focus ever more closely on the nature of larvae, and especially on potential biological products that they might naturally create.

Re-centering surgical scientific attention from wounds and onto maggots drew on contemporary medical interest in the malleability and improvability of nature. Perhaps most dramatically in the US, the discovery of hormones as critical to bodily function had fostered hope that bodily ageing might be slowed or even reversed by endocrinological means.^58^ Prominent asepsis advocate and co-developer of the eponymous Carrel-Dakin method Alexis Carrel was himself fascinated with the possibility of maximizing the capacities of living bodies, a dream that motivated his work on tissue culture and organ transplantation.^59^ Experiments aimed at uncovering processes of organic inheritance similarly appeared as potential means of transcending existing limits on agricultural production.^60^ Transforming maggots into surgically appropriate beings was an entirely plausible goal in this context. Belief in the corporeally transformative capacities of science thereby helped constitute a new site of contact between medical practice and biological research. Despite — or perhaps because of — widespread interest in insects as disease vectors, few medical practitioners of the pre-war era had contemplated the possibility that insects might be manipulated into assisting healing directly. Yet by the mid-1930s, USDA scientist William Robinson was reporting on the “present therapeutic uses of insects,” which he noted included mosquito-borne malariotherapy and German “bee venom therapy” as well as surgical maggot therapy, all “undreamed of 25 years ago.”^61^

## The Maggot Business at the USDA

Interest in harnessing entomological nature in the interests of human health overlapped with contemporary concern with the remaking of the American environment to serve economic ends. Hoover and his acolytes enacted a deeply productivist conception of nature, centered around the idea that natural resources that remained unused constituted economic “waste” in the same way as idle workers.^62^ Their dual concerns with on the one hand minimizing the role and size of the American state and on the other maximizing the economic efficiency of American nature were nevertheless neither consistent nor necessarily compatible. Some of the largest reform efforts directed toward nature of the Hoover era, such as the proposed industrialization of the Tennessee Valley, culminated in an expansion of state activity rather than its reduction. So when as a result of Veterans' Committee interest maggot cultivation became the subject of a dedicated research program at the USDA's Bureau of Entomology, the effects were ambiguous. On the one hand, the newly instantiated maggot therapy industry came to be boosted by a governmental program dedicated to its assistance. Yet the very same program would have the effect of excluding Baer's original vision, along with the organisms associated with it, from surgical practice.

Links between Hoover's project of state-facilitated economic development and the emergence of maggot research at the USDA were in fact surprisingly direct. One of the most celebrated of the scientific projects associated with Hoover during the 1920s (and a key element of the eventual Tennessee Valley scheme) had been the effort to develop what the chemist S.C. Lind called a “twentieth century version of beating the sword into a plowshare” via the conversion of explosives manufacturing capacity into that for nitrogen fixation.^63^ The Fixed Nitrogen Research Laboratory in Washington, DC became key to replicating German chemical synthesis technology which, because of its importance in munitions production, had been a major source of American strategic military anxiety during war. Originally part of the Army's Ordnance Division, the program had been incorporated into the USDA in 1921. Its subsequent success was signaled by the readiness of Hoover-connected administrators to provide chemical manufacturers such as DuPont and American Cyanamid with free access to government patents, alongside fertilizer samples and even government-made reaction catalysts. In addition to fostering a commerce-friendly environment within the USDA, nitrogen fixation research also helped cultivate broad-ranging scientific expertise. As the project wound down during the late 1920s, one of its scientists — physicist Sebastian Karrer — helped Baer design a “maggot incubator” which could automatically control temperature and humidity.^64^ Since responsibility for the continuation of Baer's research work was transferred to the USDA Bureau of Entomology's office in downtown DC following his unexpected death in 1931, this incubator may well have constituted the initial capacity for rearing maggots there. Regardless, the Bureau could offer a wealth of experience in insect cultivation, not least through its numerous programs directed at the utilization of insects to reduce crop pests.^65^

Unusually for the Bureau, their long-term appointment for maggot therapy research was not an established entomologist, but rather William Robinson, a one-time member of the University of Chicago with a background in “surgical shock” research.^66^ Whilst the connection between shock and maggot cultivation may seem obscure, Robinson had in fact used insects in his experimental studies of the phenomenon.^67^ Traumatic, or “wound” shock had moreover played a critical role during and immediately after the war in the conceptualization of wounds as having potentially whole-body as well as localized effects. No less than Henry Dale and Walter Cannon had drawn on problems relating to sepsis (especially blood sepsis) and gas gangrene infection to characterize the wounded body as a vulnerable set of interacting systems thrown out of balance by severe physiological disruption.^68^ So Robinson not only possessed an appropriate background working with insects, but also may well also have been perceived as someone who could carry the torch of holistic wound therapy for the celebrated but now departed Baer. In any case, the Bureau assigned him the tasks both of investigating maggot cultivation, and elucidating the precise means by which maggots facilitated healing.

By the time Robinson arrived in post in 1931, a nascent cottage sterile maggot industry — no doubt in part encouraged by the apparent prospect of government assistance — had begun to emerge amongst the surgical community. Approaches to cultivation varied widely: rearing techniques depended largely on the extent to which surgeons considered it necessary to guarantee maggots were microbe-free. For example, those with the greatest faith in the infection-reducing capacities of larvae characterized aseptic rearing as neither essential nor economically justifiable. Hence a trio of University of Pittsburgh-based investigators noted that their treatment of “gas bacillus infections… with grossly unsterile larvae” had achieved “amazing results.”^69^ Nevertheless, surgeons for the most part followed Baer in his attempt to exclude pathogens from larvae completely.^70^ As one reporter put it, the “modern surgeon” considered “the idea of putting into a wound anything that has not been sterilized…abhorrent.”^71^ Yet Baer's process of immersing eggs in disinfectant was labor intensive, resulted in increased larval mortality, required constant bacteriological testing, and necessitated an initial outlay of capital that the effects of the recent nationwide economic slump often made prohibitive. Moreover, from a governmental point of view, leaving responsibility for rearing endless small-scale sterile fly colonies in the hands of entomologically inexpert surgeons seemed both inefficient and unwise. Safety concerns weighed particularly heavily on administrators' minds: blowfly larvae were easily mistaken for those of the Texas screw-worm, which was known to bore through healthy as well as putrid flesh to often devastating results.^72^ Senator Johnson thus worried during Baer's Committee hearing that “without proper equipment and… training, many deaths will result.” It was it seemed desirable that the practice be guided by an appropriate, government-sanctioned body.^73^

Guaranteeing that maggots reared by surgeons were free of wound-polluting microbes thereby became an priority for the new Bureau program. Initially, government scientists focused on maximizing the cultivation efficiency of small-scale producers. Much surgical entomological effort was devoted to reducing maggot rearing costs whilst minimizing the possibility of microbial ingression into breeding colonies. Thrift remained a key concern: one orthopedic surgeon recommended manufacturing fly cages by re-using “milk-strainer wire” stretched between strips of sponge rubber.^74^ Another advertised a technique involving recycling maggots directly from wounds to form new egg-laying fly stock, allowing “one worker [to] rear enough maggots to treat some fifty patients at one time.”^75^ Yet others concentrated on maximizing the efficiency of egg sterilization. Alternatives to Baer's bichloride of mercury for this process included hydrogen peroxide, formaldehyde mixed with sodium hydroxide, sodium chloride, and even Carrel-Dakin solution itself.^76^ Robinson advocated collaboration between surgeons and the Bureau in such studies, and toured hospitals around DC giving instruction and advice on efficient breeding technique.^77^ By 1935, he was suggesting that in areas with multiple surgical institutions in close proximity, collaborative or “club rearing” might provide “a ready source of supply and at reduced costs.”^78^ His assistant Samuel W. Simmons conducted evaluations of the different proposed egg disinfectants, concluding that the highest “hatch rate” could be achieved using a formalin and sodium hydroxide mix.^79^

Yet from an entomological point of view, it was by no means clear whether such whole-organism disinfection was either effective, or indeed even possible. The problem lay in a longstanding debate amongst bacteriologists concerning the role of microorganisms in development. From 1885, a dispute had emerged in France between Émile Duclaux and Louis Pasteur regarding the dispensability or otherwise of microbes to larger forms of life. Duclaux and his followers' contention that microbial beings were invariably harmful to larger animals had contrasted with Pasteur and his supporters' claim that intestinal microbes were critical to the digestive process. Increasingly sophisticated experiments came to be conducted in an attempt to decide the question. From the mid-1890s, when the Berlin-based George H.F. Nuttall and Hans Thierfelder attempted to raise microbe-free guinea pigs, a gradually expanding range of animals had been grown in laboratory conditions excluded as far as possible from environmental contact with microscopic organisms. Species included rabbits (Kijanizin, 1895), tadpoles (O. Metchnikoff, 1902; Wollmann, 1913), chickens (Schottelius, 1902; Cohendy, 1912), mosquitoes (Atkin and Bacot, 1917; Northrup, 1926) and even goats (Küster, 1912).^80^ Flies became a notable addition to the list in 1906-1907, when blowfly larvae were similarly enrolled in studies aimed at proving the veracity of one or another of Duclaux and Pasteur's positions — first when University of Geneva professor Emile Guyenot contended that their digestion was entirely microbially dependent, and later when Pasteur Institute entomologist Eugène Wollman insisted on the contrary that it was indeed possible to raise blowflies microbe-free.^81^

Advocates of surgical maggot sterilization thereby assumed that only one amongst a range of entomological views as to the possibility of creating microbe-free maggots had prevailed.^82^ Though surgeon-conducted studies generally glossed over the fact, their entomologist collaborators remained anxious that colony-grown maggots might not in fact be as sterile as widely assumed. Ottis R. Causey, later of the Rockefeller virus laboratory in Belem, Brazil, worried that despite his utmost precautions, his own experimentally grown larvae were routinely found to harbor “both spore-forming bacilli and cocci.”^83^ Others expressed concern that the means by which sterility was guaranteed were unfit for purpose. According to the Pittsburgh study, conducting bacteriological tests by culturing a sample of disinfected eggs, as Baer and others had done, could not guarantee maggot sterility. It was it argued instead necessary to adopt the more stringent method of culturing a mixture of developed maggots and their foodstuffs. These more thorough examinations indicated that microbes might be detected as “the rule rather than the exception.”^84^ Researchers at Ohio State University were even more pessimistic, noting that maggots raised using thoroughly sterilized meat “did not mature at all.” When properly carried out, the food sterilization process caused the loss of a “food factor” associated with the production of the enzyme invertase, which was critical to development.^85^

Bacteriological and entomological anxieties thereby militated against Robinson's efforts to encourage local cultivation. Moreover, repeated admonishment that all cultivation should be left to those with specific training in entomology and bacteriology undoubtedly discouraged many prospective breeders. To those with neither the relevant expertise nor faith enough in the healing capacities of unsterilized maggots, a system by which sterile maggot provision could be guaranteed came to be seen as essential. Indeed, in an effort to allay practitioner's qualms, the Bureau itself initially offered to supply its own specimens to surgeons. As Robinson advertised, there was “*No Danger of Parasitism*… with the [blowfly] species *Lucilia sericata*” he supplied.^86^ Yet it was never the commercially-minded Bureau research directors' intention that the department become a final authority on maggot cleanliness, and even less to potentially place the USDA in competition with private medical concerns. Rather, they sought to funnel research towards the support of profit-making enterprise.

As commercial interest in surgical maggot production grew, the thrust of Robinson's collaborative efforts shifted from supporting small-scale cultivators to maximizing the breeding efficiency and distribution capacity of large firms. Lederle's initial announcement described their new product as “marketed in bottles containing approximately 1,000” larvae: surgical maggots would they promised be sent out “according to a schedule which is designed to insure [sic] an active product.”^87^ The following year, Petrolager Research Laboratories of Chicago began their own operation.^88^ Lederle produced studies emphasizing the novelty and significance of the therapy, along with the technical requirements of sterile maggot production — moves which likely helped overcome the qualms of some surgeons regarding larval cleanliness.^89^ A collaborative study involving assistant director of Lederle Edward Roberts highlighted the complexities involved in maggot rearing, arguing that contrary to the six to eight distinct wound “implantations” recommended by Baer, at least eight and sometimes up to thirty were required.^90^ Despite such puffery, Lederle representatives had by 1934 become disillusioned as to the commercial viability of their breeding facility. Robinson and Simmons conducted a field trip to the company that year, during which they were given to understand that the maggot boom was in decline as sales had slowed. Yet Simmons noted that in their view uptake amongst the medical community had been “growing rapidly,” a claim bolstered by the contemporary proliferation of research publications on the subject. Lederle's problem was rather that their quoted six-dollar-a-bottle retail price was “excessively high” due to the “unnecessarily cumbersome, inefficient, and expensive” rearing techniques in use there.^91^ Should his and Robinson's recommendations for improvements be carried out, it would lead to “lower priced maggots which naturally will make them more available” to surgeons.^92^ Robinson boasted in a report of that year that Bureau advice to Lederle would reduce commercial maggot prices “by 50%.”^93^

The most obvious manifestation of the emerging alliance between Lederle and the Bureau of Entomology concerned the problem of maggot transportation. For any large-scale maggot production facility to be viable it was necessary to ship to all parts of the US. However, maggots grew rapidly. Without some method of slowing down larval metabolism, most bottled maggots would arrive at their destination either too mature to be effective in wounds or dead. Lederle thus packed their charges in ice, claiming that this “caused them to remain…at approximately the same 2-day stage of growth.”^94^ Yet clinicians noted “considerable difference in vigor and efficiency of the various consignments” received, the fault they suggested of the “length of time involved in shipping.”^95^ In an effort to ensure the commercial viability of long-distance maggot shipment, Robinson and Simmons embarked on an intensive examination of means by which maggot development could be controlled. In 1935 Simmons thereby announced that ice transportation was no longer necessary, as a low-nutrition diet of water, evaporated milk, and agar jelly made it possible to “sustain the larvae without allowing material growth and development.”^96^ “Retardation by malnutrition,” Robinson reported to his supervisors, “could be used commercially and save a considerable part of the cost of commercial production.”^97^

Just as the popular appeal of the USDA's promise to turn bombs into fertilizer ended up as an effective subsidy of the nitrogen fixation industry, the focus of their surgical maggot program gradually turned from an effort to directly assist individual surgeons' insect cultivation efforts to an alliance with firms exploring the possibility of large-scale commercial larviculture. In collaborating with pharmaceutical manufacturers, Bureau scientists were participating in by-then orthodox governmental activity in which state-funded research would be directed first and foremost towards facilitation of corporate profit-making. Moreover, the tendency towards both economic and scientific rationalization that this focus brought with it would soon come to militate against the direct use of maggots in medicine.

## Maggots, Muck, and the Contexts of Antibiotics

As far as the pharmaceutical and organic chemistry industry of the 1930s was concerned, maggot therapy seemed virtually irrelevant. Whilst Lederle may have enjoyed a reputational boost from their breeding scheme, any income generated paled into insignificance when compared with products such as its anti-pneumococcal horse serum (based in part on principles articulated by Wright and Fleming).^98^ Moreover, Lederle had in 1930 come into the ownership of none other than American Cyanamid, the fertilizer manufacturer and beneficiary of USDA nitrogen fixation program support. In addition to the lime extract after which they had been named, the corporation had spent the 1920s using its governmentally-boosted fertilizer proceeds to diversify into the dye, sulphuric acid, pharmaceutical, and heavy chemical industries.^99^ As far as its new owner was concerned, Lederle constituted a manufacturer of medically relevant chemicals rather than a provider of medical products more generally. Commercial surgical maggot breeding came through the promotional efforts of the USDA to be enmeshed in a context intended to foster excellence in chemical synthesis rather than organic propagation. Perhaps inevitably, this circumstance in turn influenced the direction of research at the Bureau of Entomology.

Any nascent hopes for the development of an organism-centered therapeutics during the 1930s were dwarfed by medical interest in the creation of synthetic-origin substances.^100^ When it was found in 1935 that Bayer's just developed, seemingly anti-streptococcal synthetic dye derivative Prontosil in fact reduced infection due to one of the unpatented agents it broke down into (*p*-aminoben-zenesulfanilamide, or sulfanilamide), American Cyanamid's dye-making subsidiary Calco found itself in ideal circumstances for expansion into pharmaceuticals production. Working with its fellow conglomerate subsidiary Lederle, Calco founded the first sulfanilamide manufacturing plant in the US, producing around nine tons in 1937, its first year of operation.^101^ By-and-large, orthopedic surgeons and surgeon-bacteriologists fully subscribed to the assumption that the future of medical therapeutics lay in chemical synthesis or (potentially) microbiological extraction. Even many of those who acknowledged the efficacy of maggot therapy hoped that some synthetic means might be found of mimicking the effects produced by larvae. Thus even in his early outlines of potential investigative avenues, Robinson included the “possibility of isolating in tablet, capsule or other form, any part of maggot secretions or product of secretions which accelerates recovery of the patient.”^102^

Consequently, ever more attention came to be paid to the organic chemistry of maggots and their digestion-related secretions. M.A. Stewart of Houston's Rice Institute suggested for example that bone healing was due to maggot's production of calcium carbonate, which could he argued “stimulate phagocytosis.”^103^ I.H. Maseritz of the Sinai Hospital in Baltimore similarly conducted analytic studies of maggot “excreta.”^104^ But it was at the Bureau that the most careful studies of maggot exudations were made. Via minute study of the intestinal tracts of larvae, Robinson, Simmons, and their colleagues sought to identify the exact point in the digestive processes at which pathogens could no longer be detected. Their conclusions definitively supported Maseritz's suggestion that it was maggot shit, rather than orally secreted digestive substances, that held greatest bactericidal potential.^105^ Entomological study of maggot physiology thereby helped focus chemically-oriented investigation. Rather than searching for a substance intrinsic to maggot bodies, research could be concentrated on fecal matter alone.

Drawing on the extensive list of contacts built through his maggot-rearing support efforts, Robinson and his team sent out samples of maggot excrement elements that he believed might perform a significant role in the wound healing process. In return, surgeons were asked to report back on their effects. Most surgeons appear to have welcomed the chance to try out therapies that could be given to charity cases unlikely to be able to afford the longer-term hospital stay that maggot therapy itself required. Having thereby turned his breeder collaborator contact book into an ad-hoc drug trial group, Robinson began to receive reports that one extract — allantoin — had a particularly positive effect. Though this substance was well known amongst organic chemists, its potential as an antiseptic had gone almost entirely unnoticed.^106^ Robinson's write-up of his collaborator-provided cases drew considerable interest, further independent clinical trials, and eventual widespread recognition of the substance as an effective new treatment for wounds of all kinds.^107^

Indeed, likely prompted by the established trend towards investigation and utilization of biologicals, enthusiasm generated by the discovery of the healing effects of allantoin seemed to some at the Bureau to herald an entirely new horizon for wound care research.^108^ Simmons provided perhaps the most optimistic statement. In a report outlining the success of the substance, he began to articulate a broad-reaching program in which insects of all kinds might be interrogated for their bactericidal potential. “Intensive investigation” of insect enzyme physiology promised to create “a fertile field in which new substances might perhaps be isolated to cope with situations which do not yield to ordinary disinfectants.”^109^ The old synthetic antiseptics could not be relied on to combat all kinds of infection, but where they had failed, new entomologically-derived materials might fill the gaps. Moreover, allantoin's lack of toxicity seemed to some at least to give insect-sourced enzyme therapy a decided advantage over its proposed microbiological cousin. Whereas Avery and Dubos's microbial enzymes offered (albeit powerful) protection against only a narrow range of pneumococci types, the gentler, now clinically-proven (and indeed apparently even physiologically regenerative) allantoin seemed to act against many different infective organisms. Such a departure from the more technically challenging synthetic and microbiological strands of biologicals research nevertheless appeared somewhat quaint within a medical culture ever more focused on the production of bodily effects using mass produced materials. Although the *British Medical Journal* reluctantly agreed that the discovery of allantoin had proven that “maggot excrement…plays an important part in the beneficial work” of wound healing, it cautioned against over-hasty adoption of Simmons' suggestions, characterizing his anticipation of “harvesting new disinfectants possibly superior to those of non-living origin” as both “fantastic” and “unduly sanguine.”^110^ Most at the Bureau seemingly agreed. No sustained insect enzyme-centered medical research program would emerge during either Robinson or Simmons's tenures with the USDA.

Robinson and Simmons's research strategy nevertheless played to the interests of pharmaceutical and organic chemical manufacturing companies in a way that maggot rearing never could. Although allantoin had first been synthesized by Friedrich Wöhler and Justus von Liebig in 1837, it was according to Frederick R. Greenbaum of the National Drug Company, Philadelphia, only in 1935, in his own company's laboratories, that the substance was produced in any quantity.^111^ After Bureau tests seemed to prove the substance effective against “a standard strain of *Eberthella typhi*… much used in testing disinfectants,” others soon entered the market.^112^ A study of 1937 cited Merck & Co. as the principal producers of allantoin “in its commercial form,” and a Merck patent for a derivative, “bismuth allantoinate,” which “overcame” “objectionable features” of allantoin itself was filed the following year.^113^ By this time at least eight other companies had begun their own allantoin manufacturing processes, and total US production amounted to at least 150kg in 1936 alone.^114^ Pharmaceutical interest fostered belief in the possibility that allantoin might replace maggot therapy entirely. At an American Chemical Society meeting, Greenbaum reportedly “explained that synthetic chemistry had taken over the job of the maggot in healing wounds.”^115^ In 1937, Robinson proudly announced that allantoin had come “into extensive use by the medical profession” and had been incorporated into “a greaseless ointment… a cold cream… a plant jelly… [and] a combination with powdered okra.”^116^

Though pharmaceutical rhetoric might have given the impression that allantoin could completely replace maggot therapeutics, Robinson and Simmons remained acutely aware that they had only shown the substance to have beneficial effects. This was a long way from any claim it could replicate the healing effects of maggots themselves — not least given the evident role that their mechanical consumption of infected material had in the process.^117^ Moreover, the allantoin samples that they had sent out were only one amongst an increasingly wide range of chemicals identified in maggot excrement. For example, subsequent reports identified a second constituent — urea — as having beneficial wound-healing properties. These were again published in an article setting out case reports from collaborating surgeons and outlining its potential therapeutic advantages.^118^ This latter discovery seemed especially important to Robinson because it would minimize medical costs. Also first synthesized by Wohler, production of urea on a large scale had only become possible after 1922, when Carl Bosch and Wilhelm Meiser developed a means of converting ammonia produced through the fixed nitrogen process into a chemically far more stable (and therefore more widely utilizable) fertilization product. It was consequently extremely cheap. As Robinson reported, this allowed urea to be tried out on a far greater range of ailments, including “athlete’s foot and other extensive infected areas on which allantoin is not feasible because of its higher cost.”^119^ With urea, the Bureau seemed to have hit on yet another commercially viable, synthesizable medical product.

Nevertheless, in this case Robinson's suggestion encountered similar difficulties to those that had confronted advocates of maggot therapy itself. Urea was of course almost indelibly associated with urine, and therefore waste, dirt, and pollution in the American mind. Robinson appealed to practitioners and public alike to see through the association, noting that had it “first been isolated in spinach, in which it appears in considerable quantity, the name and hence the public reputation might have been far different.”^120^ There would however be no repeat of his allantoin success: professional interest remained minimal. “If the public only knew its [urea's] qualities,” he lamented in 1940, it “could be used effectively in the treatment of varicose or diabetic ulcers, heat burns, sunburn, pruritus, athletes foot, eczema, acne, lacerations, etc.”^121^ Yet lack of professional surgical interest was not necessarily a barrier to commercial efforts to establish urea as physiologically efficacious. Pharmaceutical manufacturers’ promotional efforts took a rather different tack to Robinson. Rather than confront the deep-seated cultural association of urea with urine and disease directly, they instead opted to cloak their use of the ingredient in technical jargon. By the 1940s, hand creams manufactured by Luxor and Dorothy Grey and an Armor & Co. ointment were labelled as containing carbamide — a newly-minted trade name for urea. The tactic worked. As Robinson reported in 1942, DuPont had stated that they were then selling “around 10,000lbs…for medicinal use” each month.^122^ The ingredient remains in use in beauty creams, tooth-whitening pastes and dry-skin treatments today.

Whilst it may appear somewhat coincidental that maggot principle research at the Bureau led Robinson and Simmons back towards USDA's longstanding connections with US fertilizer manufacturing, the circumstance certainly did not harm the prospects of their new maggot-inspired materia medica. Encouraged by the relative success of his recommendations, Robinson began to explore a substance found in maggot shit even more closely associated with industrial farming: ammonia itself. Though the antiseptic potential of ammonia had already been explored and rejected on the grounds that it damaged healthy cells alongside pathogenic microbes, Robinson's exhaustive commitment to the healing potential of maggot evacuations led him to more detailed studies, wherein he concluded that ammonia's antiseptic effects were due to two of its breakdown products: ammonium carbonate and ammonium bicarbonate.^123^ Though some confusion remained as to the medical efficacy of these, he became convinced of the potential of the latter.^124^ The grounds on which he stood again seemed at least economically incontrovertible. Though slightly more expensive than urea, ammonium bicarbonate was still as he commented “cheap and easily prepared for use. One-pound bottles can be purchased for about forty cents.”^125^ In the event, however, use of the substance never caught on. The sulfanilomide first manufactured on a large scale by Calco and Lederle was by this time gaining its reputation as a radically effective antibacterial agent. Whether through allantoin, urea, ammonium bicarbonate, or the sulfa- drugs, synthetic chemotherapy had it seemed finally triumphed over its briefly popular organism-centered alternative. Lederle's maggot rearing efforts appear to have been abandoned around the same time that the company began manufacturing sulfanilomide.

Yet to conclude this narrative here would be to neglect the role of maggot-based therapeutics in both the brief medical ascendancy of sulfa drugs as all-conquering magic bullets, and in the legitimation of the biological or enzyme-based antiseptics that would come to the fore under the guise of antibiotics during the 1940s. As is well known, stumbling-blocks to the widespread use of sulfas soon appeared. For example, prolonged application of sulfanilomide was found to induce toxicity, meaning it was not always appropriate for use in long-term or particularly severe infections. In addition, many practitioners found that these drugs had a tendency to dry out wounds, an effect generally recognized as unconducive to healing.^126^ With the US now fully engaged in the Second World War, allantoin seemed to offer a way around the problem. By adding this “growth-stimulating” substance to sulfanilomide powder, surgeons found the body's capacity for “active healing” to “quickly revive,” even whilst it benefited from the pathogen-destroying capacities of the synthetic chemical.^127^ The head of Robinson's division reported in 1942 that three companies had begun manufacture of sulfanilomide ointments containing additions of either allantoin, urea, or a combination of the two.^128^ Robinson himself remarked that by this time over 80 different commercial products could be associated with his maggot research.^129^ The legitimation of sulfanilomide thereby relied in part on new, maggot-derived biologicals. The maggot research-derived identification of allantoin and urea as medically efficacious in fact helped legitimate use of the very substance that would later be credited with rendering maggot therapy itself obsolete.

Further, even though the Bureau program wound down as the new war heralded tectonic shifts in US research organization and practice, the more general role of government-conducted research in the emergence of a culture of commercial drug development and exploitation in the years leading up to this point should not be forgotten.^130^ Dubos and Rolin D. Hotchkiss's 1939 announcement that they had identified a bacterially-produced enzyme sourced from soil that acted against a much wider range of microbes than previously discovered biologicals caused excitement not simply because of its quickly-confirmed efficacy, but also because of longer-term fascination with the possibility that organic beings (and indeed all agricultural substances) might constitute either fruitful sources from which anti-bacterial agents might be developed, or indeed themselves be used to combat infection.^131^ That Lederle were particularly well-placed to capitalize on the finding, becoming one of the first commercial laboratories to independently produce the substance in both quantity and quality, may well owe something to the fact that research into the antibacterial effects of fly larvae had for the preceding decade been promoted in their laboratories via a little-known government research program.^132^ Similarly, whilst it is well known that Fleming's 1927 observation that a certain strain of mold effectively destroyed Gram-positive bacteria would only be revived during the 1940s, it should also be recognized that the intervening years saw the flourishing of a promising alternative biological therapeutics focused on the healing potential of visible as well as invisible life.

## Conclusion

Neither the rapidly growing range of biologicals of the era nor the powerful synthetic antibacterial agents developed during the mid-to-late 1930s were singular precursors to the emergence of antibiotics during the 1940s. In placing the far less well remembered and seemingly esoteric practice of maggot therapy alongside the former developments, this article has brought another context for the emergence of antibiotic technologies to the fore: the in-part government sponsored interest in the inherent healing capacities of macroscopic organisms. Like that concerning biologicals, medical research and practice in this vein drew on the conceptualization of bodies as dynamic wholes. Unlike biologicals research, this strand of medical practice located medical efficacy in interactions between minimally-processed organic forms that, rather than being the preserve of major corporations, could at least in principle be produced and utilized by all. Were this approach to have triumphed, it might well have helped embody a less grandiose version of Hoover's associative state, focused on the support of small-scale industry rather than corporate monopoly. Regardless, in maggot therapy's encounter with federal research programs and corporation-oriented modes of governance, Baer and his followers' vision of simple, easily implemented insect-based wound infection control fell by the wayside. American entomological therapeutics was (at least in this case) reduced to a search for invisible biochemical substances along the lines of the well-established, technically demanding search for biologicals. This circumstance was fostered via a focused, deliberate, federally-mandated effort to reconstitute and manipulate all of nature to serve useful and commercial ends.

In detailing connections between governmental maggot breeding and both surgical and commercial activities at this time, this article has thereby identified one way in which medical culture in the US came to be influenced by Hoover and others' visions of a corporatist state. Whilst the USDA's culture of commercial collaboration may ultimately have undermined approaches to wound therapy based on the interaction of macroscopic organic bodies with one another, it should also be acknowledged that the practice itself helped foster conditions in which the healing capacities of microbial life subsequently became a matter of intense, simultaneously medical and economic concern.

## Footnotes

1 On Baer see M.M. Manring and Jason H. Calhoun, “Biographical Sketch: William S. Baer,” *Clinical Orthopaedics and Related Research* 469 (2011): 917-919.

2 Committee on World War Veterans' Legislation, *Osteomyelitis: Hearings before the Committee on World War Veterans' Legislation, House of Representatives (Seventy-First Congress). Testimony of Dr. William Stevenson Baer, April 17, 1930* (Washington: United States Government Printing Office, 1930), 1-5, 22. For context, see Beth Linker, *War's Waste: Rehabilitation in World War I America* (Chicago: University of Chicago Press, 2011).

3 Nancy Tomes, *The Gospel of Germs: Men, Women and the Microbe in American Life* (Cambridge, MA: Harvard University Press, 1999), 157-182; Naomi Rogers, “Germs With Legs: Flies Disease and the New Public Health,” *Bulletin of the History of Medicine* 63 (1989): 599-617. Whilst beyond the scope of this article, the role of metaphors relating to purity, corporeal integrity, and pollution undoubtedly played a significant role in the wider cultural context in which maggot therapy was apprehended and understood. The literature on this topic is vast. On maggots and disgust specifically, however, see Sallie Tisdale, “The Sutra of Maggots and Blowflies,” *Conjunctions* 51 (2008): 8-29.

4 Thomas Schlich, “Asepsis and Bacteriology: A Realignment of Surgery and Laboratory Science,” *Medical History* 56 (2012): 308-334; Perrin Selcer, “Standardizing Wounds: Alexis Carrel and the Scientific Management of Life in the First World War,” *British Journal for the History of Science* 41 (2008): 73-107.

5 Committee, *Osteomyelitis*, 20.

6 N.D. Bitting, “Acute Osteomyelitis and Complications,” *Southern Medical Journal* 22 (1929): 580-583, 582-583.

7 Committee, *Osteomyelitis*, 7-8.

8 Ella Maude Salmonsen, *List of References on Maggot Therapy (Reference List No. 35)* (Chicago: John Crerar Library, 1936). Despite the greater number of long-term wounded in European hospitals, maggot therapy remained largely the preserve of US surgeons during the 1930s. Consideration of the reasons for such an apparent discrepancy must however be left to a separate study. On the prior and contemporary use of maggots in non-Western approaches to wound care see, Clermont Grantham-Hill, “Preliminary Note on the Treatment of Infected Wounds with the Larva of Wohlfartia Nuba,” *Transactions of the Royal Society of Tropical Medicine and Hygiene* 27 (1933): 93-98; Hyman I. Goldstein, “Maggots in the Treatment of Infected Wounds, Complicated Fractures, Osteomyelitis and Tuberculosis Abscesses,” *Annals of Surgery* 93 (1931): 953-955.

9 See especially Ronald A. Sherman, “Maggot Therapy Takes Us Back to the Future of Wound Care: New and Improved Maggot Therapy for the 21st Century,” *Journal of Diabetes Science and Technology* 3 (2009): 336-344. Interest in other whole-organism therapeutics such as leeches may have contributed this revival. See Robert G.W. Kirk and Neil Pemberton, *Leech* (London: Reaktion, 2013), 166-175.

10 Alexander von Schwerin, Heiko Stoff, and Bettina Wahrig, ed., *Biologics: A History of Agents Made from Living Organisms in the Twentieth Century* (London: Pickering & Chatto, 2013).

11 John E. Lesch, *The First Miracle Drugs: How the Sulfa Drugs Transformed Medicine* (Oxford: Oxford University Press, 2007), 269-292; Robert Bud, *Penicillin: Triumph and Tragedy* (Oxford: Oxford University Press, 2007), 23-53; Hannah Landecker, “Antimicrobials Before Antibiotics: War, Peace, and Disinfectants,” *Palgrave Communications* 5 (2019): 11. My use of terms such as “antiseptic,” “aseptic” and “antibacterial” adhere as closely as possible to contemporary usage.

12 On biological control see Richard C. Sawyer, “Monopolizing the Insect Trade: Biological Control in the USDA, 1888-1951,” *Agricultural History* 64 (1990): 271-285; Richard Sawyer, *To Make a Spotless Orange: Biological Control in California* (Ames, IA: Iowa State University Press, 1996); Edward Deveson, “Parasites, Politics and Public Science: The Promotion of Biological Control in Western Australia, 1900–1910,” *British Journal for the History of Science* 49 (2016): 231-254; William R. Thompson, *The Biological Control of Insect and Plant Pests: A Report on the Organization and Progress of the Work of Farnham House Laboratory* (London: HMSO, 1930). The approach was also adopted by malariologists. See John B. Gerberich and Marshall Laird, *An Annotated Bibliography of Papers Relating to the Control of Mosquitos by the Use of Fish (Revised and Enlarged to 1965)*. World Health Organization, Document Number WHO/Mal/66.562. https://apps.who.int/iris/handle/10665/65337, 2-5.

13 Stefanos Gergulanos and Todd Meyers, *The Human Body in the Age of Catastrophe: Brittleness, Integration, Science and the Great War* (Chicago: University of Chicago Press, 2018); Christopher Lawrence and George Weisz, ed., *Greater Than the Parts: Holism in Biomedicine, 1920-1950* (Oxford: Oxford University Press, 1998).

14 Michelle Murphy, *The Economization of Life* (Durham, NC: Duke University Press, 2017); Lukas Rieppel, Eugenia Lean, and William Deringer, “Introduction: The Entangled Histories of Science and Capitalism,” *Osiris* 33 (2018): 1-24.

15 Ellis W. Hawley, “Herbert Hoover, the Commerce Secretariat, and the Vision of an ‘Associative State,’ 1921–1928,” *Journal of American History* 61 (1974): 116–140.

16 See for example, E. Richard Brown, *Rockefeller Medicine Men: Medicine and Capitalism in America* (Berkeley: University of California Press, 1979); William H. Schneider, ed., *Rockefeller Philanthropy and Modern Biomedicine: International Initiatives from World War I to the Cold War* (Bloomington, IN: Indiana University Press, 2002).

17 Harry M. Marks, *The Progress of Experiment: Science and Therapeutic Reform in the United States, 1900-1990* (Cambridge: Cambridge University Press, 1997), 52-59; Nicolas Rasmussen, “The Drug Industry and Clinical Research in Interwar America: Three Types of Physician Collaborator,” *Bulletin of the History of Medicine* 79 (2005): 50-80; Joseph M. Gabriel, “The Testing of Sanocrysin: Science, Profit, and Innovation in Clinical Trial Design, 1926–31,” *Journal of the History of Medicine and Allied Sciences* 69 (2014): 604–632; Harry M. Marks, “Revisiting ‘The Origins of Compulsory Drug Prescriptions,’” *American Journal of Public Health* 85 (1995): 109–115.

18 For a comparable case study, see Kirk and Pemberton, *Leech.*

19 Hawley, “Herbert Hoover,” 139.

20 Gergulanos and Meyers, *Human Body*, 252, 263-264.

21 Mateo J. Muñoz, “Lawrence Joseph Henderson: Bridging Laboratory and Social Life” (Unpublished PhD thesis, Harvard University, 2014), 95-122.

22 Theodore M. Brown, “George Canby Robinson and the ‘Patient as Person,’” in *Greater Than the Parts*, 135-160, 142.

23 Schlich, “Asepsis and Bacteriology”; Nicholas Whitfield, “Surgical Skills Beyond Scientific Management,” *Medical History* 59 (2015): 421-442.

24 Ana Carden-Coyne, *The Politics of Wounds: Military Patients and Medical Power in the First World War* (Oxford: Oxford University Press, 2014), 120-129.

25 On the Orr method see e.g., Hiram Winnett Orr, “Listerism Properly and Improperly Applied in the Treatment of Infections of Bones and Joints,” *American Journal of Surgery* 4 (1928): 465-474.

26 Linker, *War's Waste*, 79-97.

27 William S. Baer, “Sacro-Iliac Joint - Arthritis Deformans - Viable Antiseptic in Chronic Osteomyelitis,” *Proceedings of the International Assembly of the Inter-State Postgraduate Medical Association of North America* 5 (1929-1930): 365-372, 367.

28 Ibid., 370.

29 Ilana Llöwy, “Biotherapies of Chronic Diseases in the Inter-War Period: From Whitte's Peptone to Penicillum Extract,” *Studies in History and Philosophy of Biological and Biomedical Sciences* 36 (2005): 675-695.

30 Michael Worboys, “‘The Wright Way’: The Production and Standardization of Therapeutic Vaccines in Britain, 1902-13,” in *Evaluating and Standardizing Therapeutic Agents, 1890-1950*, ed. Cristoph Gradmann and Jonothan Simon (New York: Palgrave Macmillan, 2010), 153-173.

31 William S. Baer “The Treatment of Chronic Osteomyelitis with the Maggot (Larva of the Blowfly),” *Journal of Bone and Joint Surgery* 13 (1931): 438-475, 475.

32 Baer, “Sacro-Iliac Joint,” 370; Committee, *Osteomyelitis*, 12.

33 Baer, “Sacro-Iliac Joint,” 371.

34 “Science News: Maggots in Wounds,” *Science* 72 (1930): xii-xiv, xii; “Maggots Now Aid Doctors: Incubated in Laboratory for Scientific Healing,” *New York Times*, 1 March 1931, 4.

35 “Enzyme Therapy,” *Journal of the American Medical Association* 97 (1931): 1540-1541.

36 Baer, “Treatment of Chronic Osteomyelitis,” 440.

37 Committee, *Osteomyelitis*, 9.

38 Ibid., 9-10.

39 Ibid., 10, 22-24.

40 Ibid., 10-11.

41 J. Frank Hewitt, “Osteomyelitis: Development of the Use of Maggots in Treatment,” *American Journal of Nursing* 32 (1932): 31-38, 33.

42 Walter G. Stern, “Letter: Treatment of Osteomyelitis with Maggot ‘Active Principle,’” *Journal of the American Medical Association* 98 (1932): 1586.

43 Walton Martin and William G. Heeks, “Maggots and Osteomyelitis,” *Annals of Surgery* 96 (1932): 930-950, 944-945.

44 Philip M. Lovell, “Care of the Body,” *Los Angeles Times*, 15 November 1931, 20-23, 23.

45 Committee, *Osteomyelitis*, 22. On Livingston, see John Leo Graner, “S.K. Livingston and the Maggot Therapy of Wounds,” *Military Medicine* 162 (1997): 296-300.

46 E.S. Hall, “The Use of Maggots in the Treatment of Wounds,” *Maine Medical Journal* 23 (1932): 80-86, 80.

47 For example, see Stanton K. Livingston, “Therapeutics of Maggot Active Principle: Clinical Application in 1020 Cases,” *American Journal of Surgery* 35 (1937): 554-556.

48 Walter A. Coakley and Samuel Kline, “Progressive Postoperative Gangrene of Skin: Report of Case,” *American Journal of Surgery* 33 (1936): 287-290.

49 Joseph Buchman, “The Rationale of the Treatment of Chronic Osteomyelitis with Special Reference to Maggot Therapy,” *Annals of Surgery* 99 (1934): 251-259, 252-253. The absence of statistically rigorous large-scale studies makes it difficult to compare the efficacy of the procedure with contemporary alternatives. Recent studies do, however, indicate that they were at least in some cases likely to have been highly effective. See Jo C. Dumville, Gill Worthy, J. Martin Bland, Nicky Cullum, Christopher Dowson, Cynthia Iglesias, Joanne L. Mitchell, E. Andrea Nelson, Marta O. Soares, David J. Torgerson, VenUS II Team, “Larval Therapy for Leg Ulcers (VenUS II): Randomised Controlled Trial,” *BMJ* 338 (2009): b773.

50 William Robinson “Progress of Maggot Therapy in the United States and Canada in the Treatment of Suppurative Diseases,” *American Journal of Surgery* 29 (1935): 67-71, 67.

51 Baer, “Sacro-Iliac Joint,” 370.

52 Stanton K. Livingston, “The Therapeutic Active Principle in Maggots,” *Journal of Bone and Joint Surgery* 18 (1936): 751-756, 754.

53 “The Maggot Treatment of Osteomyelitis and Other Suppurative Infections,” *Bulletin of Lederle Laboratories* 1 (1933): 27-32, 27.

54 E.g., I.H. Maseritz “The Digestion of Bone by Larvae of *Phormia Regina*,” *Archives of Surgery* 28 (1934): 589-607, 597.

55 Morris A. Stewart, “The Therapeutic Behaviour of *Lucilia Sericata* Meig. Larvae in Osteomyelitis Wounds,” *Science* 79 (1934): 459-460.

56 Edward F. Roberts, “The Clinical Application of Blow-Fly Larvae,” *Scientific Monthly* 34 (1932): 531-536, 535.

57 Roberts, “Clinical Application,” 535.

58 See e.g., Nicolas Rasmussen, “Steroids in Arms: Science, Government, Industry and the Hormones of the Adrenal Cortex in the United States, 1930-1950,” *Medical History* 46 (2002): 299-324; Christer Nordlund, “Endocrinology and Expectations in 1930s America: Louis Berman's Ideas on New Creations in Human Beings,” *British Journal for the History of Science* 40 (2007): 83-104; Michael Pettit, “Becoming Glandular: Endocrinology, Mass Culture, and Experimental Lives in the Interwar Age,” *American Historical Review* 118 (2013): 1052-1076.

59 Hannah Landecker, “Building ‘A New Type of Body in Which to Grow a Cell’: Tissue Culture at the Rockefeller Institute, 1910-1914,” in *Creating a Tradition of Biomedical Research: Contributions to the History of the Rockefeller University*, ed. Darwin H. Stapledon (New York: Rockefeller University Press, 2004), 151-174.

60 See for example, Helen Anne Curry, *Evolution Made to Order: Plant Breeding and Technical Innovation in Twentieth-Century America* (Chicago: University of Chicago Press, 2016), 13-76.

61 William Robinson, “Some Therapeutic Uses of Insects and Their Products,” *Journal of Economic Entomology* 30 (1937): 41-48, 48.

62 Kendrick A. Clements, *Hoover, Conservation, and Consumerism: Engineering the Good Life* (Lawrence: University Press of Kansas, 2000), 75-96.

63 Timothy Johnson, “Nitrogen Nation: The Legacy of World War I and the Politics of Chemical Agriculture in the United States, 1916-1933,” *Agricultural History* 90 (2016): 209-229, 209. On broader links between the chemical industry and agriculture at this time, see Mark R. Finlay, “Old Efforts at New Uses: A Brief History of Chemurgy and the American Search for Biobased Materials,” *Journal of Industrial Ecology* 7 (2004): 33-46.

64 Committee, *Osteomyelitis*, 6.

65 Sawyer, “Monopolizing the Insect Trade.”

66 Robert McCormick, “Humble Healers,” *Collier's*, 14 June 1941, 57, 60.

67 William Robinson, “A Study of the Effect of Surgical Shock on Insects,” *Journal of Agricultural Research* 37 (1928): 743-748.

68 Gergulanos and Meyers, *Human Body*, 59-60, 350-351 (n. 160).

69 Grover C. Weil, Richard J. Simon, and Walter R. Sweadner, “Larval or Maggot Therapy in the Treatment of Acute and Chronic Pyogenic Infections,” *American Journal of Surgery* 19 (1933): 36-48, 47. See also Duncan Clark McKeever, “The Classic. Maggots in Treatment of Osteomyelitis: A Simple Inexpensive Method,” *Clinical Orthopaedics and Related Research* 466 (2008): 1329–1335.

70 Ottis R. Causey, “Sterilization and Growth of the Eggs and Larvae of the Blow-Fly,” *American Journal of Hygiene* 15 (1932): 276-286.

71 Jane Stafford, “Healing with Maggots: How Clean Larvae of the Blowfly are Carefully Bred to Destroy Germs Rather than Spread Them,” *Science News-Letter* 20 (1931): 122-124, 123.

72 Joseph Buchman and John E. Blair, “Maggots and their Use in the Treatment of Chronic Osteomyelitis,” *Surgery, Gynecology and Obstetrics* 55 (1932): 177-190.

73 Committee, *Osteomyelitis*, 1.

74 McKeever, “The Classic,” 1333.

75 Archie Fine and Howard Alexander, “Maggot Therapy: Technique and Clinical Application,” *Journal of Bone and Joint Surgery* 16 (1934): 572-582, 579-581.

76 “Council on Pharmacy and Chemistry: New and Nonofficial Remedies,” *Journal of the American Medical Association* 98 (1932): 401-402, 401; Fine and Alexander, “Maggot Therapy,” 576; F.F. Murdoch and T.L. Smart, “A Method of Producing Sterile Blowfly Larva for Surgical Use,” *United States Naval Medical Bulletin* 29 (1931): 406-417, 412.

77 William Robinson, “Monthly Report: August 1932,” 10 September 1932; “Monthly Report: November 1932,” [Dec. 1932]. Both folder 4047, Unpublished Manuscripts and Reports, 1926-1942. Record Group 310: Records of the Agricultural Research Service, 1906–1998. US National Archives (USNA 310).

78 Robinson, “Progress of Maggot Therapy,” 69.

79 Samuel W. Simmons, “Sterilization of Blowfly Eggs in the Culture of Surgical Maggots for Use in the Treatment of Pyogenic Infections,” *American Journal of Surgery* 25 (1935): 140-147, 144.

80 Robert G.W. Kirk, “'Life in a Germ-Free World: Isolating Life from the Laboratory Animal to the Bubble Boy,” *Bulletin of the History of Medicine* 86 (2012): 237-275, 242.

81 Emile Guyenot, “L'appareil digestif et la digestion de quelques larves de mouches,” *Bulletin scientifique de la France et de la Belgique* 41 (1907): 353-370; Eugène Wollman, “Sur l'élevage des Mouches stériles,” *Annales de l'Institut Pasteur* 25 (1911): 79-88.

82 Maseritz, “Digestion of Bone,” 598.

83 Causey, “Sterilization and Growth,” 279-280.

84 Weil, Simon, and Sweadner, “Larval or Maggot Therapy,” 40.

85 F. Fletcher and J.G. Haub, “Digestion in Blowfly Larvae. *Phormia regina* Meigen Used in the Treatment of Osteomyelitis,” *Ohio Journal of Science* (1933): 101-109, 106.

86 William Robinson, “Surgical Maggots in the Treatment of Infected Wounds, Culture of Sterile Maggots,” *Journal of Laboratory and Clinical Medicine* (1932): 406-412, 411, emphasis in the original.

87 “Council on Pharmacy,” 401.

88 “What’s New in Science: Maggot Surgery,” *Los Angeles Times*, 15 January 1933, 17.

89 Roberts, “Clinical Application,” 533.

90 Frank S. Child and Edward F. Roberts, “The Treatment of Chronic Osteomyelitis with Live Maggots,” *New York State Journal of Medicine* 15 (1931): 937-943, 939, 942.

91 Samuel W. Simmons, “Report of a Trip for the Investigation of Commercial Production of Surgical Maggots,” April 1934, folder 4215, USNA 310. For the full range of publications, see Salmonsen, *List of References on Maggot Therapy.*

92 Simmons, “Report of a Trip.”

93 William Robinson, “Quarterly Progress Report: April, May and June 1934,” 9 July 1934, folder 4279, USNA 310.

94 Child and Roberts, “Treatment of Chronic Osteomyelitis,” 939.

95 Samuel Ayres, Nelson Paul Anderson, and G. Mosser Taylor, “Maggot Therapy in Dermatologic Practice: Report of a Case of Chronic Ulcerating Granuloma of Undetermined Ethology in which Maggot Therapy Was Used,” *Archives of Dermatology and Syphilology* 33 (1936): 21-30, 28.

96 Samuel W. Simmons, “Adequacy of Nutritional Retardation in Culture of Sterile Maggots for Surgical Use,” *Archives of Surgery* 30 (1935): 1024-1031, 1025.

97 William Robinson, “Monthly Report: Sept. 1932,” [Oct. 1932], folder 4157, USNA 310.

98 Arturo Casadevall and Matthew D. Scharff, “Serum Therapy Revisited: Animal Models of Infection and Development of Passive Antibody Therapy,” *Antimicrobial Agents and Chemotherapy* 38 (1994): 1695-1702, 1698-1699.

99 Paula Kepos, ed., *International Directory of Company Histories*, Vol. 8 (London: St. James Press, 1994), 24-26, 24.

100 Rasmussen, “Steroids in Arms.”

101 Anthony S. Travis, *Dyes Made in America 1915-1980: The Calco Chemical Company, American Cyanamid, and the Raritan River* (Jerusalem: Hexagon, 2004).

102 William Robinson, “Research Plan: Relation of Fly Maggots to Osteomyelitis,” 15 June 1931, folder 3954, USNA 310.

103 Stewart, “Therapeutic Behavior,” 460.

104 Maseritz, “Digestion of Bone,” 589-607, 594.

105 William Robinson and Vernon H. Norwood, “Destruction of Pyogenic Bacteria in the Alimentary Tract of Surgical Maggots,” *Journal of Laboratory and Clinical Medicine* 19 (1934): 581-586.

106 Frederick R. Greenbaum, “Allantoin: A New Granulation Tissue Stimulating Substance with Especial Emphasis on Allantoin in Ointment Form,” *American Journal of Surgery* 34 (1936): 259-265, 259.

107 For example, Theodore Kaplan, “The Allatonin Treatment of Ulcers,” *Journal of the American Medical Association* 108 (1937): 968-969.

108 “Allantoin Hailed as Effective Drug: Success in Treating Burns is Proved by Its Wide Use, Government Says,” *New York Times*, 15 June 1936, 13.

109 Samuel W. Simmons “A Bactericidal Principle in Secretions of Surgical Maggots,” *Journal of Bacteriology* 30 (1935): 253-267, 266.

110 “How Surgical Maggots Act,” *British Medical Journal* 2 (1935): 1108-1109.

111 Greenbaum, “Allantoin,” 260.

112 Simmons, “Bactericidal Principle,” 256.

113 Kaplan, “Allatonin Treatment,” 968; “New Agent Speeds Healing in Wounds,” *New York Times*, 16 January 1938, 47.

114 Fred C. Bishopp, “Annual Report for Uniform Projects,” 13 June 1936, folder 4438, USNA 310; William Robinson, “Quarterly Progress Report: Jan., Feb. and March 1937,” 9 April, 1937, folder 4786, USNA 310.

115 “Chemists, Doctors End Controversy: Secret Debate at Pittsburgh Brings an Accord for More Tests on Seydel Drug – New Gains for Allantoin,” *New York Times*, 12 September 1936, 15.

116 William Robinson, “Quarterly Progress Report: Jan., Feb. and March 1937,” 9 April 1937, folder 4786, USNA 310.

117 William Robinson, “The Stimulation of Healing in Non-Healing Wounds,” *Journal of Bone and Joint Surgery* 17 (1935): 267-271, 269.

118 William Robinson, “Use of Urea to Stimulate Healing in Chronic Purulent Wounds,” *American Journal of Surgery* 33 (1936): 192-197, 196.

119 William Robinson, “Quarterly Progress Report: Jan., Feb. and March 1936,” 8 April 1936, folder 4615, USNA 310.

120 McCormick, “Humble Healers,” 60; Robinson, “Use of Urea,” 197.

121 William Robinson, “Quarterly Progress Report: Jan., Feb. and March, 1940,” 6 April 1940, folder 6092, USNA 310.

122 William Robinson, “Annual Progress Report,” [January] 1942, folder 5340, USNA 310.

123 William Robinson, “Ammonia as a Cell Proliferant and its Spontaneous Production from Urea by the Enzyme Urease,” *American Journal of Surgery* 49 (1940): 319-325.

124 Fred C. Bishopp, “Annual Report for Uniform Projects,” [June] 1938, folder 4438, USNA 310.

125 William Robinson, “Ammonium Bicarbonate Secreted by Surgical Maggots Stimulates Healing in Purulent Wounds,” *American Journal of Surgery* (1940): 111-115, 114.

126 Lesch, *First Miracle Drugs*, 171-181.

127 J. Ross Veal, Roy G. Klepser, and Michael DeVito, “The Preparation of Superficial Wounds for Skin Grafting by the Local Use of Sulfanilamide and Sulfanilamide-Allantoin Ointment,” *American Journal of Surgery* (1941): 716-720, 717.

128 Fred C. Bishopp, “Annual Report for Uniform Projects,” 4 June 1942, folder 4438, USNA 310.

129 William Robinson, “Annual Progress Report,” c. January 1942, folder 5340, USNA 310.

130 The last extant report is William Robinson, “Annual Progress Report: Investigations of the Secretions of Insects and their Effects on Man and Animals, 1942,” [Dec] 1942, folder 5397, USNA 310.

131 Jill E. Cooper, “A Brief Romance with Magic Bullets: René Dubos at the Dawn of the Antibiotic Era,” in *Silent Victories: The History and Practice of Public Health in Twentieth Century America*, ed. John W. Ward and Christian Warren (Oxford: Oxford University Press, 2007), 44-59. 46-50.

132 Rollin D. Hotchkiss, “From Microbes to Medicine: Gramicidin, René Dubos, and the Rockefeller,” in *Launching the Antibiotic Era: Personal Accounts of the Discovery and Use of the First Antibiotics*, ed. Carol L. Moberg and Zanvil A. Cohn (New York: Rockefeller University Press, 1990), 1–18.

